# Seroprevalence of anti-SARS-CoV-2 IgG antibodies in HIV-positive and HIV-negative patients in clinical settings in Douala, Cameroon

**DOI:** 10.3389/fepid.2023.1212220

**Published:** 2023-08-14

**Authors:** Sylvie Kwedi Nolna, Miriam Niba, Cedric Djadda, Palmer Masumbe Netongo

**Affiliations:** ^1^ Epidemiology Department, Faculty of Medicine and Biomedical Sciences, University of Yaoundé 1, Yaoundé, Cameroon; ^2^ Capacity for Leadership Excellence and Research (CLEAR), Yaoundé, Cameroon; ^3^ Department of Biochemistry, Faculty of Sciences, University of Yaoundé 1, Yaoundé, Cameroon; ^4^ Molecular Diagnostics Research Group, Biotechnology Centre-University of Yaounde I, Yaoundé, Cameroon; ^5^ School of Science, Navajo Technical University, Crownpoint, NM, United States

**Keywords:** COVID-19, SARS-CoV-2, IgG antibodies, HIV, Cameroon

## Abstract

**Background:**

The asymptomatic nature of COVID-19 coupled with differential testing are confounders in the assessment of SARS-CoV-2 incidence among people living with HIV (PLWH). As various comorbidities increase the risk of SARS-CoV-2 infection, it is crucial to assess the potential contribution of HIV to the risk of acquiring COVID-19. Our study aimed to compare the anti-SARS-CoV-2 IgG seroprevalence among people living with and without HIV.

**Methods:**

PLWH were enrolled in the HIV units of two health facilities in Douala, Cameroon. Participants were consecutively enrolled, among which 47 were people living with HIV and 31 were HIV-negative patients. SARS-CoV-2 antibody tests were performed on all participants. Overall, medical consultation was conducted. For HIV-positive participants only, viral load, antiretroviral regimen, duration of HIV infection, and duration of antiretroviral treatment were retrieved from medical records.

**Results:**

We found an overall SARS-CoV-2 IgG seroprevalence of 42.31% within the study population, with a SARS-CoV-2 IgG seroprevalence of 44.6% for PLWH and 38.7% among those without HIV infection; no significant statistical difference was observed. Adjusting for sex, HIV status, and BCG vaccination, the odds of previous SARS-CoV-2 infection were higher among married persons in the study population. Sex, BCG vaccination, and HIV status were not found to be associated with SARS-CoV-2 IgG seropositivity.

**Conclusions:**

Our findings support the lack of association between HIV status and susceptibility to SARS-CoV-2 infection. The ARV regimen, suppressed viral load, and Tenofovir boasted ARV regimen might not affect the body’s immune response after exposure to SARS-CoV-2 among PLWH. Thus, if HIV is well treated, the susceptibility to COVID-19 in PLWH would be like that of the general population.

## 1. Introduction

Since early 2020, the COVID-19 pandemic has affected the world in an unprecedented manner. The impact of this disease on people living with HIV (PLWH) is still being explored. Reports from various studies show a lower incidence of COVID-19 among PLWH (0%–3.7%) compared to the general population (4%–7.4%) (1–4). It thus may appear that HIV patients with normal CD4 T-cell counts, suppressed viral loads, and receiving regular combined antiretroviral therapy (cART) do not present with a severe clinical course of COVID-19 and may not be at an increased risk of developing SARS-CoV-2 infection (4). However, the high asymptomatic nature of COVID-19 coupled with differential testing has been reported to be confounders in the assessment of SARS-CoV-2 incidence among PLWH compared to the general population. According to an analysis conducted in San Francisco, matching the San Francisco Department of Public Health COVID-19 testing and case database and the San Francisco Department of Public Health HIV Surveillance case registry, PLWH were found to be more susceptible to COVID-19 infection compared to the general population (5). In South Africa, a setting of relatively high HIV endemicity, it was found that, due to an HIV-related immune response, PLWH may experience more severe disease from COVID-19 (6).

PCR-based testing is still a major challenge in most parts of Africa and might be the reason why PCR-based SARS-CoV-2 prevalence data is scarce. Besides, there is a high degree of variability in the incidence of COVID-19 in African communities compared to those of other continents. This warrants seroprevalence studies to determine actual exposure rates within the general population and representative subsets, such as PLWA. Despite the challenge of conducting clinical research during an ongoing pandemic, a few systematic seroprevalence studies comparing SARS-CoV-2 infection rates by HIV status have been conducted in Italy, the USA (7, 8), and some parts of Africa (9, 10), suggesting fewer SARS-CoV-2 infections among PLWH compared to the general population. Like the rest of Africa, Cameroon is affected by COVID-19 and HIV pandemics counting over 504,472 PLWH with a prevalence of 2.9% in adults aged 15–49 years (11). The country registered its first confirmed COVID-19 case on 4 March 2020. As of 10 March 2023, 1,965 deaths and 124,392 COVID-19 cases were recorded in Cameroon (12). In a recent study conducted in Cameroon, SARS-CoV-2 antibodies were found in PLWH as early as 2019, which may attest to the presence of the disease before the pandemic was declared (13). Since various comorbidities increase the risk of SARS-CoV-2 infection, it is crucial to assess the potential contribution of HIV to the risk of acquiring this new pathogen. With the ongoing debate on whether PLWH might be at an increased risk of severe COVID-19 (13), our study, which used serological testing, will contribute to the emerging data measuring the COVID-19 disease burden in PLWH. Understanding seroprevalence and the rate of actual cases is necessary to better manage epidemics, hospitalizations, and deaths in a well-defined population, helping to make some inferences about a larger population. Our study aimed to compare the anti-SAR-CoV-2 IgG seroprevalence among people living with and without HIV receiving healthcare in two non-governmental health facilities in Douala, Cameroon.

## 2. Materials and methods

### 2.1. Study design

Using a cross-sectional study design, patients were recruited from the Adlucem Clinic Bonaberi and the Adlucem Clinic Bonamoussadi, two non-governmental health facilities in Douala, from 1 October 2021 to 28 February 2022. PLWH were enrolled as they attended the HIV units of both facilities. Simultaneously, HIV-negative patients were recruited from the same health facility's outpatient unit after a negative HIV screening test. After obtaining voluntary consent, participants were subjected to a SARS-CoV-2 antibody test. In addition, sociodemographic, clinical, and HIV-related information (for PLWH) was collected through a study-developed questionnaire. Participants considered for this study were those: with a known HIV history and followed up at the HIV unit, who were randomly selected (for the HIV group) or tested HIV-negative at the external consultation (for the HIV negative group); aged 21 years or older; residing in the town of Douala for the last 12 months; willing to take a COVID-19 screening test; willing to take part in the study; and those who signed an informed consent form.

### 2.2. Sample size calculations

The sample size was estimated to detect a 40% probability of having COVID-19 antibodies among the general population, with absolute precision of 5% at a 95% confidence level. A total of 78 participants were selected consecutively, among which 47 were people living with HIV and 31 were people living without HIV.

The sample size was calculated using OpenEpi software version 3.01 based on a method proposed by Kelsey et al., for the calculation of sample size for an unmatched case-control study (14). The following assumptions were made: a two-sided confidence level(1-alpha) = 95, power (% chance of detecting) = 80, the ratio of Controls to Cases = 1, the hypothetical proportion of controls with exposure = 40, the hypothetical proportion of cases with exposure:16.5, and least extreme Odds Ratio to be detected: 0.30. This study targeted to enroll 132 participants: 66 HIV-positive participants and 66 HIV-negative participants. In all, 149 participants were screened, among which 74 were PLWH.

### 2.3. SARS-CoV-2 IgG and viral load measurement

Detection of SARSCoV-2 IgG was done using lateral flow immunochromatographic assay Wondfo® (Guangzhou Wondfo Biotech Co., China). Wondfo® SARS-CoV-2 IgG antibody test is an immunochromatographic assay for rapid and quantitative detection of SARSCoV-2 IgG antibody in human biological samples. The assay has a sensitivity of 95.6% and a specificity of 98.4%, as reported by the manufacturer (15). A blood sample of 5 ml was used for SARS-CoV-2 antibody characterization. For HIV-positive participants, an additional blood sample was taken from participants whose last viral load control result dated more than 6 months.

### 2.4. Clinical and sociodemographic data

Medical conditions such as chronic disease history, viral load, antiretroviral regimen, duration of HIV infection, and duration of antiretroviral treatment were assessed via consultation of participants’ medical records for PLWH and HIV-negative patients, and a medical consultation was conducted. In addition, a patient-administered questionnaire was used to collect sociodemographic data.

### 2.5. Statistical analysis

Mobile-based KoBo toolbox software (https://kf.kobotoolbox.org/#/forms) was used to collect and manage data, which was later extracted into an Excel sheet for verification, cleaning, and validation. Participants lacking a verifiable HIV or SARS-CoV-2 status were not included in the analysis. The validated data was then transferred into a newly created database in the SPSS, R, and Stata software for appropriate analysis. Descriptive statistics were performed using SPSS. The intergroup comparisons of SARS-CoV-2 IgG seropositivity were done by performing a test of proportions using R. Categorical variables, on the other hand, were presented in rates or proportions and compared amongst them using the chi-square test. The risk factors of disease acquisition in the two groups were determined using bivariate logistic regression, which was later adjusted for each other's effect in a multivariate logistic regression from which the independent risk factors were identified. The significance of all the above tests was set at 5%.

## 3. Results

### 3.1. Demographic characteristics of the study population

As shown in Table 1, of the 78 participants enrolled in the study, 47 were living with HIV, while 31 were HIV-negative. The median age was 35 ± 11 years for PLWH and 40 ± 11 years for the non-HIV group. There was no observed difference in religion, clinical history, marital status, and smoking status across the two population groups. Within the HIV-infected group, 34 (72%) were women, among whom two were pregnant. In the HIV-negative group, 41.9% of persons were obese (BMI ≥ 30 kg/m^2^), while the majority of the PLWH (57.4%) were of average weight (BMI between 18.5 and 24.9 kg/m^2^) with no obesity documented. Most of the participants within the HIV group had received a BCG vaccine at birth (97.9%), had a suppressed viral load (61.7%), and were on a TLD (Tenofovir + Lamuvidine + Dolutegravir) antiretroviral regimen. No participant had reported a previous COVID-19 infection, and no one was vaccinated against SARS-CoV-2.

**Table 1:** Demographic characteristics (*N* = 78).

** **	** **	PLWH	HIV negative	*p-value*
Sex	Male	13 (27.7%)	18 (58.1%)	0.007
	Female	34 (72.3%)	13 (41.9%)	
Age group (years)	21–30	15 (31.9%)	13 (41.9%)	0.05
	31–45	23 (48.9)	16 (51.6%)	
	45–70	08 (17%)	12 (38.7%)	
Marital status	Single	32 (68.1%)	17 (54.8%)	0.114
	Married	10 (21.3%)	13 (41.9%)	
	Living together	4 (8.5%)	0	
	Widow(er)	1 (2.1%)	1 (3.2%)	
Body mass index	Underweight	2 (4.3%)	0	0.005
	Normal weight	27 (57.4%)	7 (22.6%)	
	Overweight	12 (25.5%)	10 (32.3%)	
	Obese	0	13 (41.9%)	
Pregnancy status	Pregnant	2 (5.9%)	0	0.019
	Not pregnant	32 (94.1%)	13 (100%)	
History of chronic disease history	Pulmonary disease	2 (4.3%)	1 (3.2%)	0.65
	Cardiovascular disease	1 (2.1%)	2 (6.5%)	0.346
	Diabetes	1 (2.1%)	0	0.63
Smoking status	Smoker	2 (4.3%)	1 (3.2%)	0.65
	Non-smoker	45 (95.7%)	30 (96.8%)	
BCG vaccination	Vaccinated	46 (97.9%)	18 (58.1%)	0.000
	Not vaccinated	1 (2.1%)	13 (41.9%)	
HIV duration (months)	Less than 12	15 (31.9%)		
	More than 12	30 (63.8%)		
Viral load	Unsuppressed	6 (12.8%)		
	Suppressed	29 (61.7%)		
ART regimen	TLD	42 (89.4%)		
	TELE (TDF-lamivudine, efavirenz)	5(10.6%)		

### 3.2. SARS-CoV-2 IgG seropositivity among PLWH and HIV-negative populations

Among the 78 participants, SARS-CoV-2 IgG antibodies were detected in 33, giving a SARS-CoV-2 IgG seroprevalence of 42.31% within the study population. The SARS-CoV-2 IgG seroprevalence was 44.6% (21 out of 47) among people with HIV and 38.7% (12 out of 31) among those without HIV infection. No statistical difference was observed between the seroprevalence of the two study groups at a 95% confidence interval.

### 3.3. Factors associated with SARS-CoV-2 IgG positivity

As shown in Table 2, adjusting for sex, HIV status, and BCG vaccination status within both study groups, the odds of previous SARS-CoV-2 infection (SARS-CoV-2 IgG positive) were 80% higher among married persons. Sex, BCG vaccination, and HIV status were not found to be associated with SARS-CoV-2 IgG seropositivity. Among the group of PLWH, an HIV duration of more than 12 months, an unsuppressed viral load (>40 copies/ml), and receiving TLD (Tenofovir + Lamuvidine + Efavirenz) as antiretroviral treatment was not found to be associated with SARS-CoV-2 IgG seropositivity.

**Table 2:** Factors associated with SARSCoV-2 IgG positivity.

** **	Odd ratio	*P*-value	95% CI
Full cohort			
Male	0.13	0.85	0.32–4.0
Married	1.85	0.001***	0.97–19.7
Living together	0.65	0.69	0.08–49.31
Widow(er)	1.27	0.33	0.27–46.16
HIV	0.79	0.63	0.09–54.1
BCG vaccination	0.39	0.61	0.33–6.62
People living with HIV			
Male at birth	0.04	0.97	0.12–9.07
Unsuppressed viral load (>40 copies/ml)	0.61	0.8	0.016–217.44
More than 12 months of HIV infection	0.03	0.98	0.04–25.25
Receiving TLD	1.84	0.2	0.37–105.62

*** *p* < 0.01.

## 4. Discussion

In this study, we described the seroprevalence of SARS-CoV-2 IgG among people with and without HIV receiving healthcare in two non-governmental health facilities in Douala, Cameroon. The general SARS-CoV-2 IgG seroprevalence in this study was estimated at 42.31% and was relatively higher among PLWH, at 44.68% (21/47), compared to HIV-negative patients, at 38.71%, though no statistical significance was observed. A previous community-based study in Cameroon reported a 29.2% seroprevalence of anti-SARSCoV-2 IgG antibodies (16). It should be noted that this previous investigation occurred from 14 October to 26 November 2020, a year earlier in the pandemic when compared to the present study. Other studies in different parts of the world have documented evidence of previous exposure to SARS-CoV-2. In Iran, for example, an IgG seroprevalence of 39.0% was found in a study conducted at the beginning of the pandemic, in April and May 2020 (17).

We note some variability in reported seroprevalence results in Africa ranging from 2.6% in Sierra Leone (18) to 45.1% in Nigeria (19). In our study population, participants did not report any previous SARS-CoV-2 infection, no one had received a COVID-19 vaccine, and all were asymptomatic. It is thus possible that the IgG seropositivity observed within this population indicates the participants’ natural immune response to SARS-CoV-2 infection, irrespective of their HIV status. Several reasons, such as the nature of the virus, the host’s characteristics, and various aspects of the African environment, were previously evoked for the unexpectedly low burden of COVID-19 reported in Africa since the beginning of the pandemic (20). Taking only the pre-vaccine period into account, levels of SARS-CoV-2 IgG seroprevalence around the world were also found to vary. In a study conducted in the United States, a seroprevalence of 20.2% was found during the period of July 2020–May 2021 (21). In Canada, also during the pre-vaccine period, a prevalence of 4.6% was found (22), while in Brazil around the same period, a seroprevalence of 25.4% was found (23). For the eastern region of the world, a study conducted in Kuwait found a seroprevalence of 24.8% (24). In more general terms, a meta-analysis was conducted on articles from 88 countries, namely, original articles published from December 2019 to December 2021, and it was concluded that the pooled estimate of seroprevalence SARS-CoV-2 was 15% in Eastern Mediterranean countries, 6% in Africa, 8% in the Americas, 5% in Europe, and 3% in the Western Pacific (25). It is challenging to discuss comparisons with such a variety of results.

Underreporting has also been claimed to be responsible for the lower COVID-19 disease burden in Africa compared to other parts of the world (26–28). Our study corroborates this claim as 42% of our participants who showed evidence of previous infection were never reported as confirmed cases of COVID-19. In addition, the asymptomatic nature of the participants indicates a mild form of COVID-19 occurrence within the population, as found in several studies around the world (29–32). Mindful of the unimmunized nature of the study population, this asymptomatic nature could be the result of a pre-existing cross-reactive immunity conferred by the exposure of the Cameroonian population to other forms of coronaviruses prior to the onset of the pandemic, as suggested by Aissatou et al. (13). Furthermore, relative higher seroprevalence (although not statistically significant) was recorded among PLWH compared to the HIV-negative group. Meanwhile, HIV-positive patients were on a Tenofovir antiretroviral regimen, which could mean that PLWH with adequate adherence to ART (antiretroviral treatment) are not at a lower risk of contracting COVID-19 compared to the general population. Similar results were found in a documented case series of 33 patients in German HIV centers where darunavir and Tenofovir ARV boasted regimens were shown to increase the risk of SARS-CoV-2 infection in HIV patients (33).

As shown in Table 2, when adjusted for age, sex, and religion, the odds of having suffered from a previous infection were significantly associated with marriage. In a previous seroprevalence study conducted in Cameroon by Fai et al., age, gender, and comorbidities were significantly associated with SARS-CoV-2 IgG antibodies, contrary to our findings (34). These findings may be attributed to the difference in the study populations. In addition, the Fai study included hospitalized patients admitted with severe COVID-19 disease, which may explain the association between age and comorbidities.

In our study, among the PLWH, suppressed viral load, ART regimen, and duration of HIV infection were not significantly associated with IgG seropositivity. Our findings are similar to those obtained in a study conducted in Spain, where no association was found between suppressed viral load and SARS-CoV-2 seropositivity among PLWH (8). The possible protective benefits of Tenofovir on SARS-CoV-2 infection are likely due to its immunomodulatory effects (35). Other studies refuted the assumption that PLWH under a Tenofovir regimen and suppressed viral load are at a lower risk of COVID-19 morbidity and mortality. Nomah et al., found that PLWH receiving tenofovir alafenamide (TAF) and tenofovir disoproxil fumarate (TDF) were not at a higher risk of SARS-CoV-2 infection (36). At the same time, another study failed to find excess morbidity or mortality among PLWH, especially those with viral load suppression on ART (10).

### 4.1. Limitations of the study

A matched case-control study design may have improved the efficiency in determining the strength of the association between HIV status and the presence of SARS-CoV-2 IgG antibodies. Unfortunately, matching was not feasible as our participant recruitment abilities were constrained by the health facilities' patient attendance, resulting in a high refusal rate. In Cameroon, the legal age of majority to be able to consent is 21 years. Any adults aged 18–20 were thus automatically excluded, limiting the results' ability to generalize to the general adult population. All the PLWH recruited in this study were on a tenofovir ARV boasted regimen. It will be interesting to investigate the effect of other non-tenofovir ARV regimens.

## 5. Conclusion

Our findings support the lack of association between HIV status and susceptibility to SARS-CoV-2 infection. Furthermore, as shown in previous investigations, the ARV regimen, suppressed viral load, and Tenofovir boasted ARV regimen might not affect the body's immune response after exposure to SARS-CoV-2 among PLWH and HIV-negative individuals. Thus, if HIV is well treated, the susceptibility to COVID-19 would be similar to those of the general population.

Future studies with larger samples, including multiple study sites and subgroup analyses based on the immunological status of HIV-positive patients, will provide further evidence.

## Data Availability

The datasets generated during and/or analyzed for study are available in a data repository through the following link (https://redcap.mrc.gm:8443/redcap/redcap_v12.0.20/ProjectSetup/index.php?pid=298).

## References

[1] del Amo J Polo R Moreno S Díaz A Martínez E Arribas JR Incidence and severity of COVID-19 in HIV-positive persons receiving antiretroviral therapy a cohort study. Ann Intern Med. (2020) 173(7):1–5. 10.7326/M20-3689 32589451 PMC7394316

[2] Tesoriero JM Swain CAE Pierce JL Zamboni L Wu M Holtgrave DR COVID-19 outcomes among persons living with or without diagnosed HIV infection in New York state. JAMA Netw Open. (2021) 4(2):1–11. 10.1001/jamanetworkopen.2020.37069 PMC7859843 33533933

[3] Inciarte A Gonzalez-Cordon A Rojas J Torres B de Lazzari E de la Mora L Clinical characteristics, risk factors, and incidence of symptomatic coronavirus disease 2019 in a large cohort of adults living with HIV: a single-center, prospective observational study. AIDS. (2020) 34(12):1775–9. 10.1097/QAD.0000000000002643 32773471 PMC7493771

[4] Huang J Xie N Hu X Yan H Ding J Liu P Epidemiological, virological and serological features of coronavirus disease 2019 (COVID-19) cases in people living with human immunodeficiency virus in Wuhan: a population-based cohort study. Clin Infect Dis. (2021) 73(7):1–16. 10.1086/339445 32803216 PMC7454403

[5] Patel RH Acharya A Mohan M Byrareddy SN. COVID-19 and AIDS: outcomes from the coexistence of two global pandemics and the importance of chronic antiretroviral therapy. J Med Virol. (2021) 93(2):641–3. 10.1002/jmv.26416 32779740 PMC7436736

[6] Sachdev D Mara E Hsu L Scheer S Rutherford G Enanoria W COVID-19 susceptibility and outcomes among people living with HIV in San Francisco. J Acquir Immune Defic Syndr. (2021) 86(1):19–21. 10.1097/QAI.0000000000002531 33044323 PMC7727319

[7] Papalini C Paciosi F Schiaroli E Pierucci S Busti C Bozza S Seroprevalence of anti SARS-CoV2 antibodies in umbrian persons living with HIV. Mediterr J Hematol Infect Dis. (2020) 12(1):e2020080. 10.4084/mjhid.2020.080 33194154 PMC7643777

[8] Spinelli MA Lynch KL Yun C Glidden D V Peluso MJ Henrich TJ SARS-CoV-2 seroprevalence, and IgG concentration and pseudovirus neutralising antibody titres after infection, compared by HIV status: a matched case-control observational study. Lancet HIV. (2021) 8(6):e334–41. 10.1016/S2352-3018(21)00072-2 33933189 PMC8084354

[9] Boulle A Davies MA Hussey H Ismail M Morden E Vundle Z Risk factors for coronavirus disease 2019 (COVID-19) death in a population cohort study from the western cape province, South Africa. Clin Infect Dis. (2021) 73(7):2005–13. 10.1093/cid/ciaa1198 PMC7499501 32860699

[10] Kanwugu ON Adadi P. HIV/SARS-CoV-2 coinfection: a global perspective. J Med Virol. (2021) 93(2):726–32. 10.1002/jmv.26321 32692406 PMC7404432

[11] UN Office for the Coordination of Humanitarian Affairs. Cameroon ReliefWeb. Cameroon: COVID 19 Emergency Situation Report (SITREP) No. 22—1 January to 28 February 2022 (2022).

[12] Center for Systems Science and Engineering (CSSE) at Johns Hopkins University (JHU). Covid-19 dashboard. Covid-19 Resource Center. (2022). Available at: https://coronavirus.jhu.edu/region (Accessed June 20, 2022). 10.1016/S1473-3099(22)00434-0 PMC9432867 36057267

[13] Aissatou A Fokam J Semengue ENJ Takou D Ka’e AC Ambe CC Pre-existing immunity to SARS-CoV-2 before the COVID-19 pandemic era in Cameroon: a comparative analysis according to HIV-status. Front Immunol. (2023) 14:1–6. 10.3389/fimmu.2023.1155855 PMC10116492 37090738

[14] Kelsey JL Whittemore AS Evans AS Thompson WD. Table 12–15. In: Methods in observational epidemiology. Oxford, United Kingdom: Oxford University Press (1996). p. 432.

[15] Boum Y Fai KN Nikolay B Mboringong AB Bebell LM Ndifon M Performance and operational feasibility of antigen and antibody rapid diagnostic tests for COVID-19 in symptomatic and asymptomatic patients in Cameroon: a clinical, prospective, diagnostic accuracy study. Lancet Infect Dis. (2021) 21(8):1089–95. 10.1016/S1473-3099(21)00132-8 33773618 PMC7993929

[16] Nwosu K Fokam J Wanda F Mama L Orel E Ray N SARS-CoV-2 antibody seroprevalence and associated risk factors in an urban district in Cameroon. Nat Commun. (2021) 12(1):1–8. 10.1038/s41467-021-25946-0 34615863 PMC8494753

[17] Balou HA Yaghubi Kalurazi T Joukar F Hassanipour S Shenagari M Khoshsorour M High seroprevalence of SARS-CoV-2 (COVID-19)-specific antibodies among healthcare workers: a cross-sectional study in Guilan, Iran. J Environ Public Health. (2021) 2021:2–6. 10.1155/2021/9081491 PMC8536443 34691195

[18] Barrie MB Lakoh S Kelly JD Kanu JS Squire JS Koroma Z SARS-CoV-2 antibody prevalence in Sierra Leone, March 2021: a cross-sectional, nationally representative, age-stratified serosurvey. BMJ Glob Health. (2021) 6(11):1–5. 10.1136/bmjgh-2021-007271 PMC8587532 34764148

[19] Olayanju O Bamidele O Edem F Eseile B Amoo A Nwaokenye J SARS-CoV-2 seropositivity in asymptomatic frontline health workers in Ibadan, Nigeria. Am J Trop Med Hyg. (2021) 104(1):91–3. 10.4269/ajtmh.20-1235 33185181 PMC7790104

[20] Tcheutchoua DN Tankeu AT Angong DLW Agoons BB Nguemnang NYY Djeunga HCN Unexpected low burden of coronavirus disease 2019 (COVID-19) in Sub-Saharan Africa region despite disastrous predictions: reasons and perspectives. Pan Afr Med J. (2020) 37:1–9. 10.11604/pamj.2020.37.352.25254 PMC7992902 33796166

[21] Jones JM Stone M Sulaeman H Fink R V Dave H Levy ME Estimated US infection- and vaccine-induced SARS-CoV-2 seroprevalence based on blood donations, July 2020–May 2021. JAMA. (2021) 326(14):1400–7. 10.1001/jama.2021.15161 34473201 PMC8414359

[22] Charlton CL Nguyen LT Bailey A Fenton J Plitt SS Marohn C Pre-vaccine positivity of SARS-CoV-2 antibodies in Alberta, Canada during the first two waves of the COVID-19 pandemic. Microbiol Spectr. (2021) 9(1):1–10. 10.1128/Spectrum.00291-21 PMC8552659 34406813

[23] Hallal PC Hartwig FP Horta BL Silveira MF Struchiner CJ Vidaletti LP SARS-CoV-2 antibody prevalence in Brazil: results from two successive nationwide serological household surveys. Lancet Glob Health. (2020) 8(11):1390–6. 10.1016/S2214-109X(20)30387-9 PMC7511212 32979314

[24] Alfouzan W Altawalah H AlSarraf A Alali W Al-Fadalah T Al-Ghimlas F Changing patterns of SARS-CoV-2 seroprevalence: a snapshot among the general population in Kuwait. Vaccines (Basel). (2023) 11(2):2–11. 10.3390/vaccines11020336 PMC9963614 36851214

[25] Azami M Moradi Y Moradkhani A Aghaei A. SARS-CoV-2 seroprevalence around the world: an updated systematic review and meta-analysis. Eur J Med Res. (2022) 27:1–32. 10.1186/s40001-022-00710-2 35655237 PMC9160514

[26] Okonji EF Okonji OC Mukumbang FC Van Wyk B. Understanding varying COVID-19 mortality rates reported in Africa compared to Europe. Americas and Asia. Trop Med Int Health. (2021) 26(7):716–8. 10.1111/tmi.13575 33733568 PMC8251241

[27] Mwananyanda L Gill CJ Macleod W Kwenda G Pieciak R Mupila Z COVID-19 deaths in Africa: prospective systematic postmortem surveillance study. BMJ. (2021) 372:1–10. 10.1136/bmj.n334 PMC7887952 33597166

[28] Dutschke A Wejse C Nanque JP Medina C Hønge BL Jespersen S SARS-CoV-2 seroprevalence among people living with HIV in Guinea–Bissau. Public Health. (2022) 209:26–38. 10.1016/j.puhe.2022.05.017 PMC9157020 35785597

[29] Tanaka H Ogata T Morisada K Tanaka S Yoshida T Nakanishi H Transmission of SARS-CoV-2 through contact with a SARS-CoV-2-infected individual in the presymptomatic or asymptomatic state in Japan: a case series. Nihon Koshu Eisei Zasshi. (2021) 68(8):556–8. 10.11236/jph.20-145 33994491

[30] Johansson MA Quandelacy TM Kada S Prasad PV Steele M Brooks JT SARS-CoV-2 transmission from people without COVID-19 symptoms. JAMA Netw Open. (2021) 4(1):1–7. 10.1001/jamanetworkopen.2020.35057 PMC7791354 33410879

[31] El-Ghitany EM Hashish MH Farghaly AG Omran EA Osman NA Fekry MM. Asymptomatic versus symptomatic SARS-CoV-2 infection: a cross-sectional seroprevalence study. Trop Med Health. (2022) 50(1):1–11. 10.1186/s41182-022-00490-9 36575501 PMC9792933

[32] Rodeles LM Peverengo LM Benítez R Benzaquen N Serravalle P Long AK Seroprevalence of anti-SARS-CoV-2 IgG in asymptomatic and pauci-symptomatic people over a 5 month survey in Argentina. Rev Panam Salud Publica. (2021) 45(1):1–7. 10.26633/RPSP.2021.66 PMC8216497 34168682

[33] Härter G Spinner CD Roider J Bickel M Krznaric I Grunwald S COVID-19 in people living with human immunodeficiency virus: a case series of 33 patients. Infection. (2020) 48(5):682–5. 10.1007/s15010-020-01438-z PMC7211976 32394344

[34] Fai KN Corine TM Bebell LM Mboringong AB Nguimbis EBPT Nsaibirni R Serologic response to SARS-CoV-2 in an African population. Sci Afr. (2021) 12:1–7. 10.1016/j.sciaf.2021.e00802 PMC8164732 34095639

[35] Castillo-Mancilla JR Meditz A Wilson C Zheng JH Palmer BE Lee EJ Reduced immune activation during tenofovir-emtricitabine therapy in HIV-negative individuals. J Acquir Immune Defic Syndr. (2015) 68(5):1–8. 10.1097/QAI.0000000000000529 25763783 PMC4358752

[36] Nomah DK Reyes-Urueña J Díaz Y Moreno S Aceiton J Bruguera A Impact of tenofovir on SARS-CoV-2 infection and severe outcomes among people living with HIV: a propensity score-matched study. J Antimicrob Chemother. (2022) 77(8):2265–73. 10.1093/jac/dkac177 35678461 PMC9384242

